# Supplementary figures and images for: Ionized acrylamide-based copolymer / terpolymer hydrogels for recovery of positive and negative heavy metal ions
Source: PLoS One. 2024 Mar 1;19(3):e0298047. doi: 10.1371/journal.pone.0298047 (PMC10906855; doi:10.1371/journal.pone.0298047)

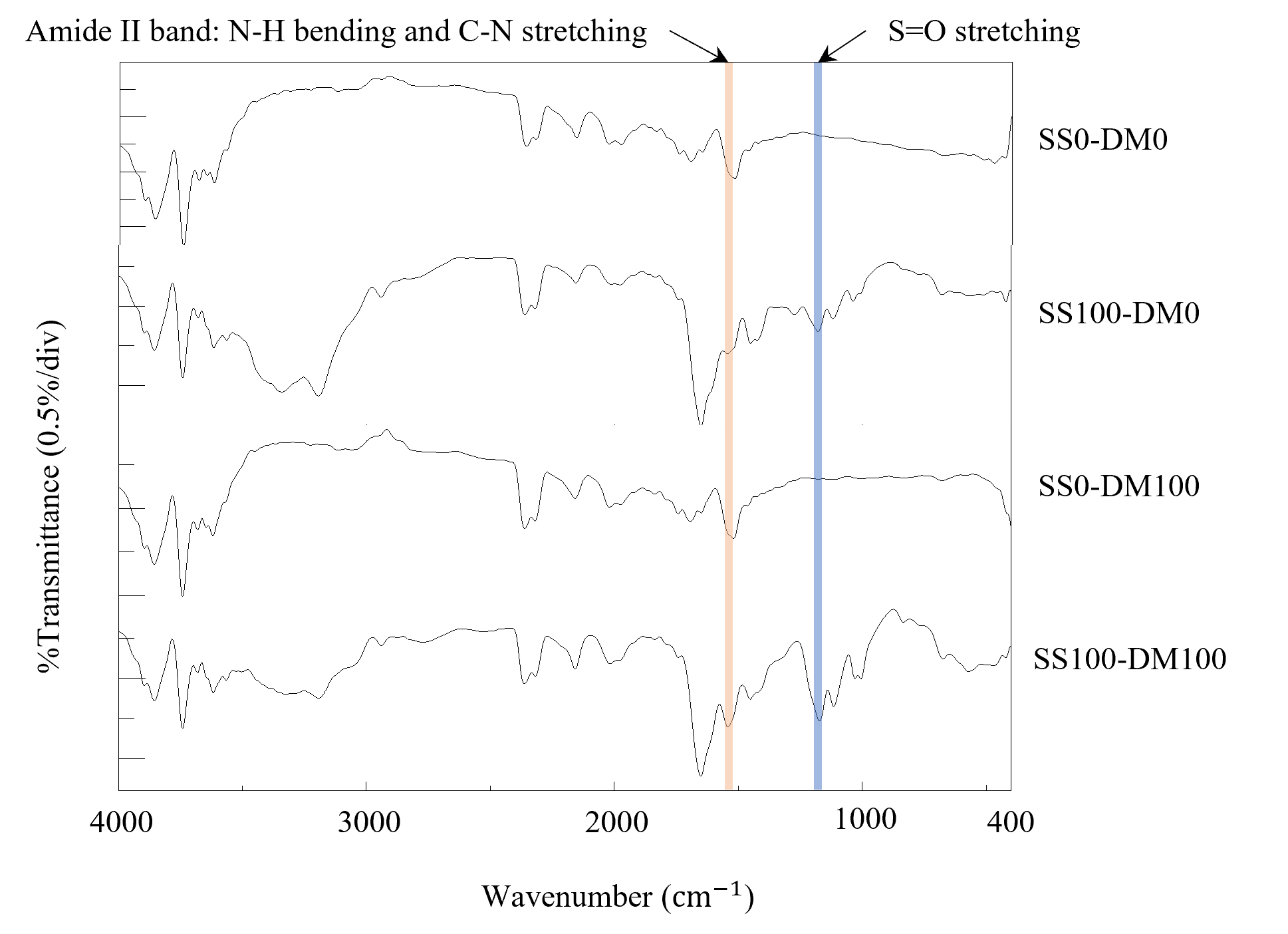

Supplement: S1 Fig — In the spectrum obtained from the gel prepared using SS, a peak corresponding to S = O stretching at a wave number of 1200–1185 cm-1 derived from SS is observed. This suggests that SS was introduced. On the other hand, a change in the amide band II at a wave number of 1550–1590 cm-1 derived from DMAPAA can be observed in the gel using DMAPAA, but this change is also due to AAm, and it also appears in the gel without DMAPAA. So, it is not possible to judge the introduction of DMAPAA. FT-IR measurements were performed using the gel samples that were dried at 60°C under pressure to make them flat. All data were measured using FT/IR-4000 with Attenuated Total Reflection (ATR) attachment (JASCO corp., Japan). (TIF) [file pone.0298047.s001.tif]

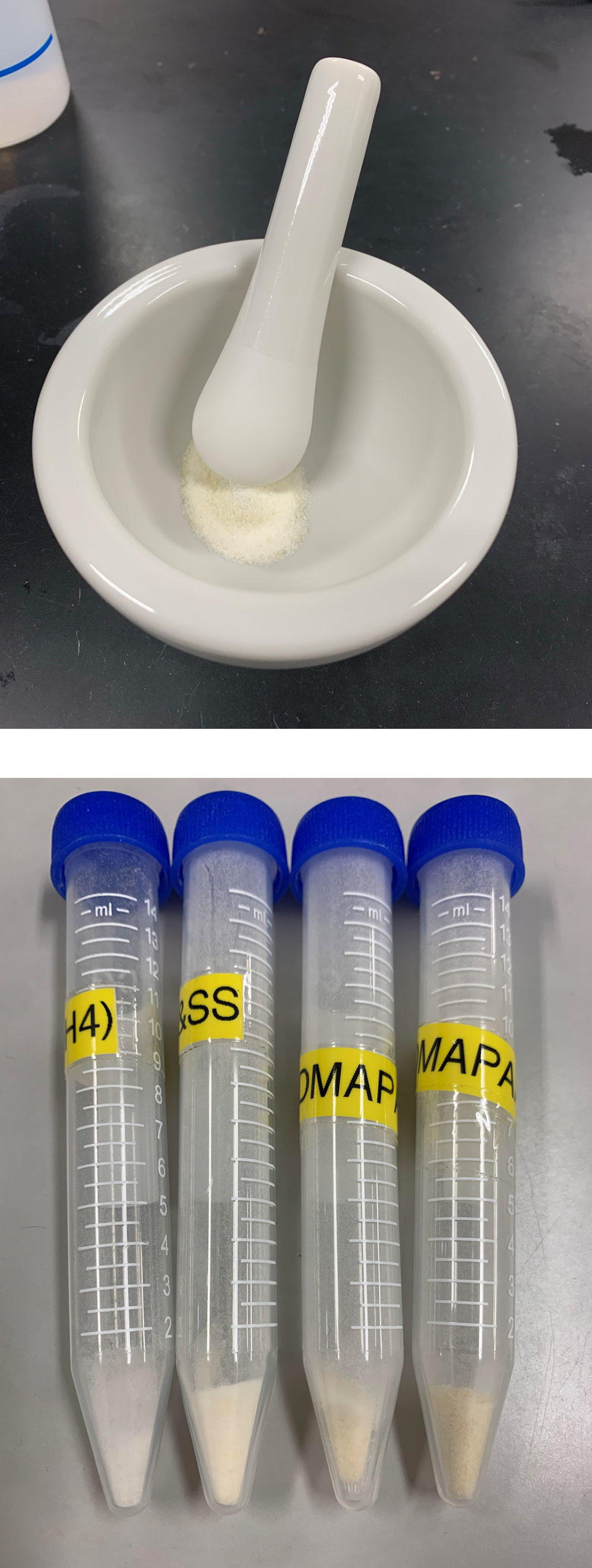

Supplement: S2 Fig — After thoroughly drying the gels, they were ground into powders in a mortar. (TIF) [file pone.0298047.s002.tif]

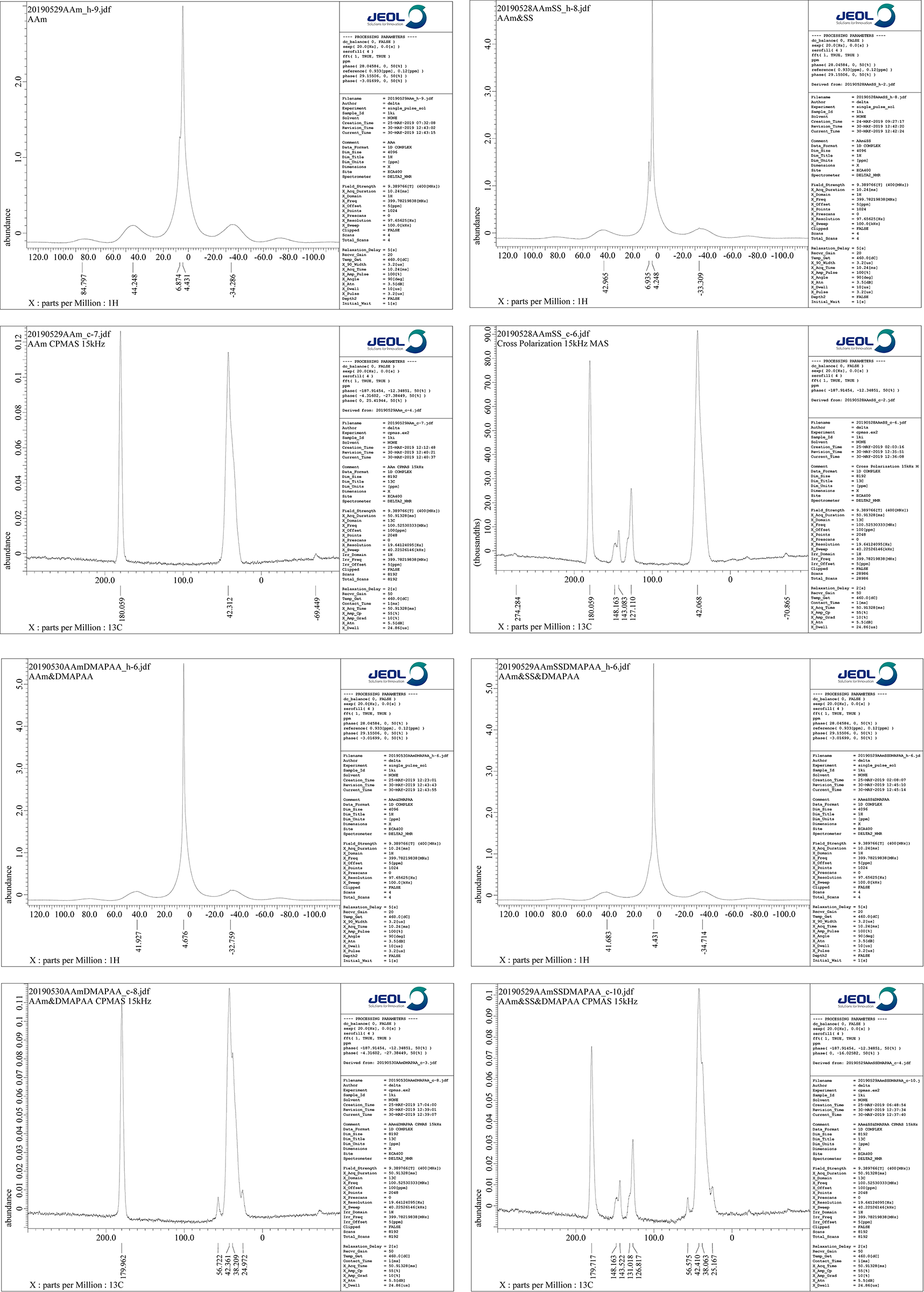

Supplement: S3 Fig — (a) 1H and 13C spectra of SS0-DM0 sample (i.e., AAm gel). (b) 1H and 13C spectra of SS200-DM0 copolymer gel. (c) 1H and 13C spectra of SS0-DM200 copolymer gel. (d) 1H and 13C spectra of SS100-DM100 terpolymer gel. The measuring conditions are shown in their right column. The 1H peaks don’t provide any useful structural information, but the 13C peaks are unique to each monomer. Please refer main part. (TIF) [file pone.0298047.s003.tif]

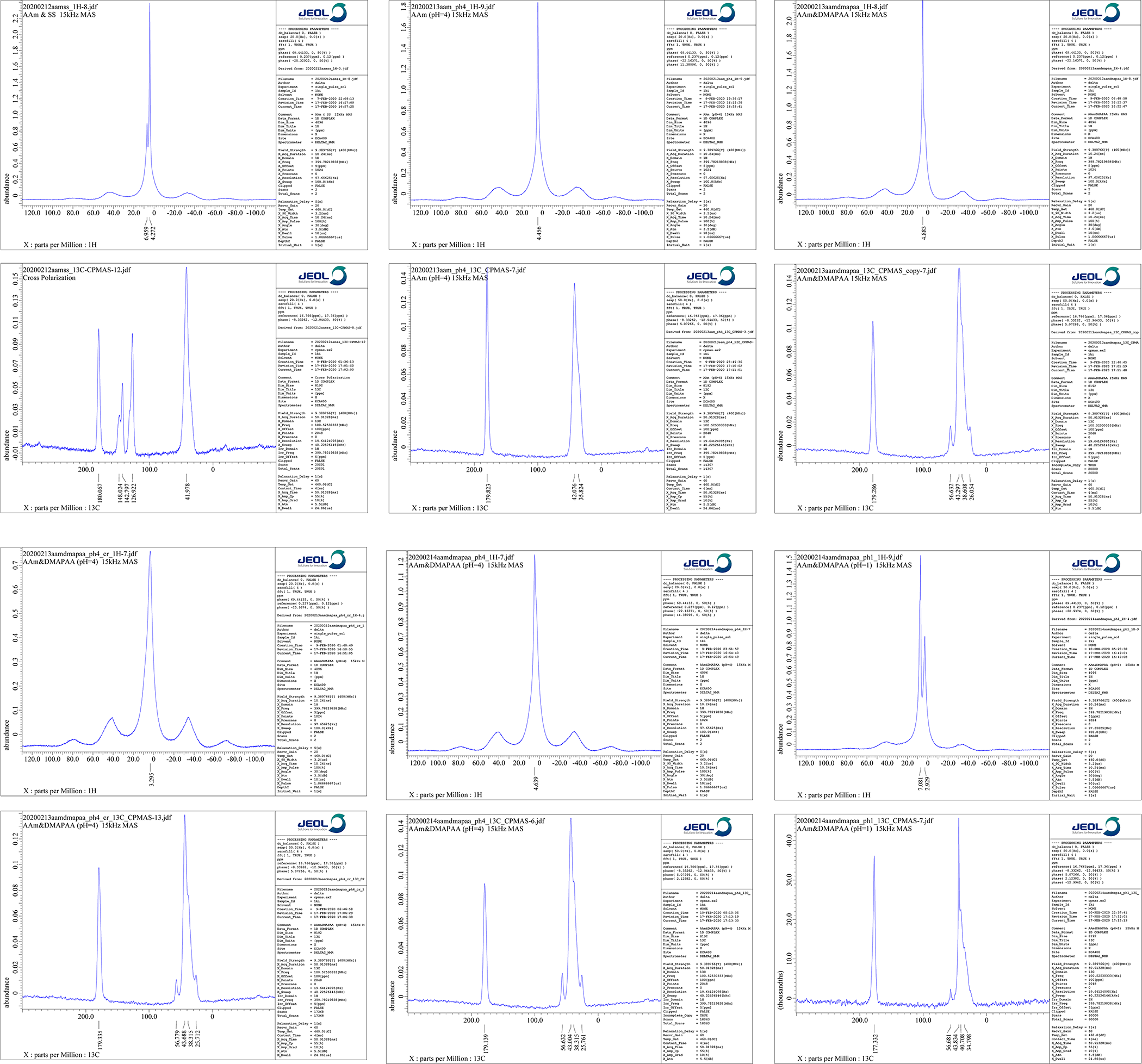

Supplement: S4 Fig — (a) 1H and 13C spectra of SS200-DM0 sample. Data obtained by re-measuring the sample with the same components as S3(A) Fig. (b) 1H and 13C spectra of SS0-DM0 sample (i.e., AAm gel) immersed in pH4 HNO3 solution for 72 hours. (c) 1H and 13C spectra of SS0-DM200 copolymer gel. Data obtained by re-measuring the sample with the same components as S3(C) Fig. (d) 1H and 13C spectra of SS100-DM100 terpolymer gel immersed in pH4 HNO3 solution with Cu2+ and (Cr2O7)2- for 72 hours. (e) 1H and 13C spectra of SS100-DM100 terpolymer gel immersed in pH4 HNO3 solution for 72 hours. (f) 1H and 13C spectra of SS100-DM100 terpolymer gel immersed in pH1 HNO3 solution for 72 hours. The measuring conditions are shown in their right column. The 13C spectra in figures (c) and (d) are used to make Fig 7. (TIF) [file pone.0298047.s004.tif]

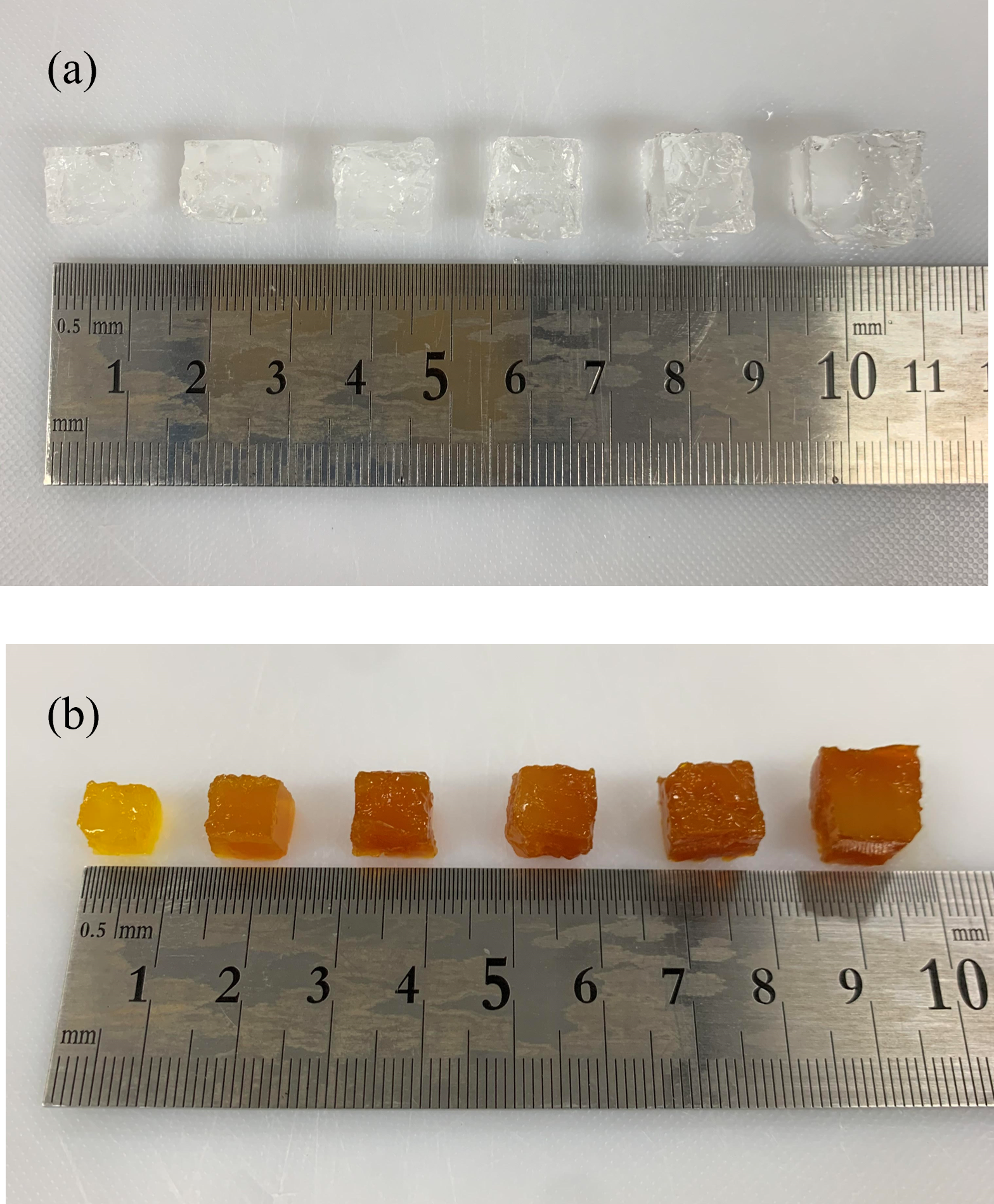

Supplement: S5 Fig — Pre-absorption (a) and post-absorption (b) profiles of AAm–DMAPAA hydrogels. Both photos show DM100, DM200, DM300, DM400, DM500, and DM600 from the left, and the higher the DMAPAA concentration, the more turbidity occurs after adsorption. It is thought that fine particles of copper hydroxide are trapped inside the gel. (TIF) [file pone.0298047.s005.tif]
