# Supplementary material for: Ionized acrylamide-based copolymer / terpolymer hydrogels for recovery of positive and negative heavy metal ions
Source: PLoS One. 2024 Mar 1;19(3):e0298047. doi: 10.1371/journal.pone.0298047 (PMC10906855; doi:10.1371/journal.pone.0298047)
Supplement: Attachment — Submitted filename: Response to Reviewers.docx [file pone.0298047.s006.docx]

------------------------------------------------------------------------

Dear Editor,

The author who originally wrote the manuscript and the author who took over this have moved on for work, and I need time to communicate with them, so the revisions have been delayed, but we appreciate your continued support. We thought it would be easier to understand if we wrote them in the email text we received, so we wrote our response after mark of ****. We have replied each item below.

Best regards,

Hirotaka Okabe and other authors

--------------------------------------------------------------

PONE-D-23-29103

Ionized acrylamide copolymer and terpolymer hydrogel absorbents for fractional recovery of positive and negative heavy metal ions from wastewater

PLOS ONE

Dear Dr. Okabe,

Thank you for submitting your manuscript to PLOS ONE. After careful consideration, we feel that it has merit but does not fully meet PLOS ONE’s publication criteria as it currently stands. Therefore, we invite you to submit a revised version of the manuscript that addresses the points raised during the review process.

Please submit your revised manuscript by Nov 24 2023 11:59PM. If you will need more time than this to complete your revisions, please reply to this message or contact the journal office at [plosone@plos.org](mailto:plosone@plos.org). When you're ready to submit your revision, log on to <https://www.editorialmanager.com/pone/> and select the 'Submissions Needing Revision' folder to locate your manuscript file.

Please include the following items when submitting your revised manuscript:

- A rebuttal letter that responds to each point raised by the academic editor and reviewer(s). You should upload this letter as a separate file labeled 'Response to Reviewers'.
- A marked-up copy of your manuscript that highlights changes made to the original version. You should upload this as a separate file labeled 'Revised Manuscript with Track Changes'.
- An unmarked version of your revised paper without tracked changes. You should upload this as a separate file labeled 'Manuscript'.

If you would like to make changes to your financial disclosure, please include your updated statement in your cover letter. Guidelines for resubmitting your figure files are available below the reviewer comments at the end of this letter.

If applicable, we recommend that you deposit your laboratory protocols in protocols.io to enhance the reproducibility of your results. Protocols.io assigns your protocol its own identifier (DOI) so that it can be cited independently in the future. For instructions see: <https://journals.plos.org/plosone/s/submission-guidelines#loc-laboratory-protocols>. Additionally, PLOS ONE offers an option for publishing peer-reviewed Lab Protocol articles, which describe protocols hosted on protocols.io. Read more information on sharing protocols at <https://plos.org/protocols?utm_medium=editorial-email&utm_source=authorletters&utm_campaign=protocols>.

We look forward to receiving your revised manuscript.

Kind regards,

Nayan Ranjan Singha, Ph.D.
Academic Editor
PLOS ONE

Journal Requirements:

When submitting your revision, we need you to address these additional requirements.

1. Please ensure that your manuscript meets PLOS ONE's style requirements, including those for file naming. The PLOS ONE style templates can be found at 
<https://journals.plos.org/plosone/s/file?id=wjVg/PLOSOne_formatting_sample_main_body.pdf> and 
<https://journals.plos.org/plosone/s/file?id=ba62/PLOSOne_formatting_sample_title_authors_affiliations.pdf>

**** We intend to follow all instructions.

2. We note that this submission includes NMR spectroscopy data. We would recommend that you include the following information in your methods section or as Supporting Information files:
a) The make/source of the NMR instrument used in your study, as well as the magnetic field strength. For each individual experiment, please also list: the nucleus being measured; the sample concentration; the solvent in which the sample is dissolved and if solvent signal suppression was used; the reference standard and the temperature.
b) A list of the chemical shifts for all compounds characterised by NMR spectroscopy, specifying, where relevant: the chemical shift (δ), the multiplicity and the coupling constants (in Hz), for the appropriate nuclei used for assignment.
c)The full integrated NMR spectrum, clearly labelled with the compound name and chemical structure.
We also strongly encourage authors to provide primary NMR data files, in particular for new compounds which have not been characterised in the existing literature. Authors should provide the acquisition data, FID files and processing parameters for each experiment, clearly labelled with the compound name and identifier, as well as a structure file for each provided dataset. See our list of recommended repositories here: <https://journals.plos.org/plosone/s/recommended-repositories>

**** We provide all original NMR data with the measuring condition in Supporting Information.

3. Thank you for stating the following in the Acknowledgments Section of your manuscript: 
   "This work was supported by JSPS KAKENHI Grant Numbers JP21656239, JP24360398, JP19H02660, JP21H01871. The NMR measurements were supported by Evaluation Center of Materials Properties and Function, Institute for Materials Chemistry and Engineering, Kyushu University."

We note that you have provided funding information that is not currently declared in your Funding Statement. However, funding information should not appear in the Acknowledgments section or other areas of your manuscript. We will only publish funding information present in the Funding Statement section of the online submission form. 
Please remove any funding-related text from the manuscript and let us know how you would like to update your Funding Statement. Currently, your Funding Statement reads as follows: 
  "This work was supported by JSPS KAKENHI Grant Numbers JP21656239, JP24360398, JP19H02660, JP21H01871. "

Please include your amended statements within your cover letter; we will change the online submission form on your behalf.

**** We would like to remove "This work was supported by JSPS KAKENHI Grant Numbers JP21656239, JP24360398, JP19H02660, JP21H01871. " from the manuscript.

4. We note that the grant information you provided in the ‘Funding Information’ and ‘Financial Disclosure’ sections do not match.

When you resubmit, please ensure that you provide the correct grant numbers for the awards you received for your study in the ‘Funding Information’ section.

**** As same as the assignment of 3.

5. Thank you for stating the following financial disclosure: 
   "This work was supported by JSPS KAKENHI Grant Numbers JP21656239, JP24360398, JP19H02660, JP21H01871. "

Please state what role the funders took in the study.  If the funders had no role, please state: "The funders had no role in study design, data collection and analysis, decision to publish, or preparation of the manuscript." 
If this statement is not correct you must amend it as needed. 
Please include this amended Role of Funder statement in your cover letter; we will change the online submission form on your behalf.

**** Please add "The funders had no role in study design, data collection and analysis, decision to publish, or preparation of the manuscript.".

Additional Editor Comments:

Comments from the Editor

1. The authors should provide at the first revision stage: Details of the NMR instrument and method used, a full list of chemical shifts, and labelled integrated copies of the NMR spectra.
**** We provide all original NMR data with the measuring condition in Supporting Information.

2. Title should be changed to the following:
Ionized acrylamide-based copolymer/ terpolymer hydrogels for recovery of positive and negative heavy metal ions
**** We change the title as suggested.
3. Both abstract and conclusion should be more focused.
**** We intend to rewrite it as instructed.
------------------------------------------------------------------------

[Note: HTML markup is below. Please do not edit.]

Reviewers' comments:

Reviewer's Responses to Questions

**Comments to the Author**

1. Is the manuscript technically sound, and do the data support the conclusions?

The manuscript must describe a technically sound piece of scientific research with data that supports the conclusions. Experiments must have been conducted rigorously, with appropriate controls, replication, and sample sizes. The conclusions must be drawn appropriately based on the data presented.

Reviewer #1: Partly

Reviewer #2: Partly

2. Has the statistical analysis been performed appropriately and rigorously?

Reviewer #1: No

Reviewer #2: N/A

3. Have the authors made all data underlying the findings in their manuscript fully available?

The [PLOS Data policy](http://www.plosone.org/static/policies.action#sharing) requires authors to make all data underlying the findings described in their manuscript fully available without restriction, with rare exception (please refer to the Data Availability Statement in the manuscript PDF file). The data should be provided as part of the manuscript or its supporting information, or deposited to a public repository. For example, in addition to summary statistics, the data points behind means, medians and variance measures should be available. If there are restrictions on publicly sharing data—e.g. participant privacy or use of data from a third party—those must be specified.

Reviewer #1: Yes

Reviewer #2: No

4. Is the manuscript presented in an intelligible fashion and written in standard English?

PLOS ONE does not copyedit accepted manuscripts, so the language in submitted articles must be clear, correct, and unambiguous. Any typographical or grammatical errors should be corrected at revision, so please note any specific errors here.

Reviewer #1: Yes

Reviewer #2: Yes

------------------------------------------------------------------------

Dear Reviewer 1,

The author who originally wrote the manuscript and the author who took over this have moved on for work, and I need time to communicate with them, so the revisions have been delayed, but I appreciate your continued support. Thank you for your valuable feedback. In consideration of your comments, we have made as many corrections as possible. We thought it would be easier to understand if we wrote them in the email text we received, so we wrote our response after mark of ****. We have replied each item below.

Best regards,

Hirotaka Okabe and other authors

------------------------------------------------------------------------

5. Review Comments to the Author

Please use the space provided to explain your answers to the questions above. You may also include additional comments for the author, including concerns about dual publication, research ethics, or publication ethics. (Please upload your review as an attachment if it exceeds 20,000 characters)

Reviewer #1: Manuscript entitled “Ionized acrylamide copolymer and terpolymer hydrogel absorbents for fractional recovery of positive and negative heavy metal ions from wastewater” submitted by Kentaro Fujimoto, Brian Adala Omondi, Shinya Kawano, Yoshiki Hidaka, Kenji Ishida, Hirotaka Okabe, Kazuhiro Hara, can be considered for publication in PLOS ONE Journal, after a serious major revisions.
Here is a list of my specific comments:
1. General comment: The novelty and practical applicability of this study should be clearly highlighted in the manuscript.
**** We intend to rewrite it as you said.
2. Page 1, Abstract: (a) Delete the first sentence. (b) Add in this section the most important experimental results to highlight the importance of this study. (c) What are the ions selected for this study???.

**** We rewrote the Abstract as suggested.

3. Page 1, Introduction: This section should be reorganized. The most important aspects related to this topic must be clearly presented to provide a properly presentation of the state of art in this field. Also, the metal ions selected for this study must be mentioned.
**** We rewrote the Abstract as suggested.

4. Page 2, line 40: “Methodologies for heavy metal wastewater…”. Add here as reference the papers: doi.org/10.1016/j.jclepro.2018.06.261 and doi.org/10.3390/polym15102301, because are relevant for this observation.
**** We added the references.
5. Page 2, line 49: “Cr(VI) is a notorious pollutant due to its…”. Add here some references.
**** We added the reference.
6. Page 3, line 75: “…selected heavy metal cations,…”. The metal ions selected for this study should be mentioned.
**** The ionic formulas were shown.
7. Page 3, line 79: “for reuse[18]–[20].”. Delete these references, because here are presented the objectives of this study.
**** We think your comments are useful, but we think it would be better to leave it as a reference for many readers.
8. Page 3, line 81: The same observation as above.
**** We repeated for emphasis. Is it better to summarize them?
9. Page 5, line 130: Replace “ICP/MS machine” with “ICP/MS equipment (or spectrometer)”.
**** We fixed.

10. Page 5, The effect of DMAPAA in (AAm – DMAPAA) copolymer hydrogels metal absorption: Some paragraphs have be already mentioned before. These should be deleted. Also, provide a clear presentation of the experimental methodology.
**** We think your points are useful, but many researchers in this field are very busy, so we think this is a useful way of writing for those who don't want to read the full text.
11. Page 7, Structural analysis using NMR: These experimental results must be more detailed discussed.
**** We have added a detailed discussion.

12. Page 7, Absorption from combined heavy metal cation/anion solutions: This section should be reorganized. All experimental results must be clearly presented and detailed discussed. Irrelevant general observations should be deleted.
**** We have moved the parts that should be written in the introduction to Introduction.

13. Page 10, Terpolymer hydrogel absorption swelling behavior: The same observations as above.

14. Page 13, Copolymer hydrogel absorption, swelling and effect of internal pH environment: The same observations.

**** Regarding points 13 and 14, We think it is necessary to show the results because they are not the same thing.
15. Page 17, Conclusion: This section must be reorganized. Delete (1), (2),…, and provide in this section a clear presentation of the most important experimental results and findings.

**** We intended to make the corrections as you told us to do.
16. Page 18, References: The number of references must be increased.
**** We added some references.

17. Fig. 8 should be moved into Supplementary materials.

**** We moved it to "Supporting information".

------------------------------------------------------------------------

Dear Reviewer 2,

Thank you for your valuable feedback. The author who originally wrote the manuscript and the author who took over this have moved on for work, and I need time to communicate with them, so the revisions have been delayed, but I appreciate your continued support. In consideration of your comments, we have made as many corrections as possible. We thought it would be easier to understand if we wrote them in the email text we received, so we wrote our response after mark of ****. We have replied each item below.

Best regards,

Hirotaka Okabe and other authors

------------------------------------------------------------------------

Reviewer #2: Dear Authors
Thank you for your interest in the PLOS ONE. I have received your manuscript (PONE-D-23-29103) entitled “Ionized acrylamide copolymer and terpolymer hydrogel absorbents for fractional recovery of positive and negative heavy metal ions from wastewater". However, after reading your paper, you should take the comments very carefully. My opinion is that the manuscript should be Reviewed again after Major revision and the manuscript should be resubmitted.

The following are comment to the author:-
1- Chemical structure of the used materials should be provided in experimental part.
**** We provide the chemical structures of the used materials in experimental part.
2- FTIR analysis should be performed for some selected prepared terpolymers and also for mono-polymers.
**** We provide the pre-liminary FT-IR data in Supporting Information.
3- All analytical devices should be mentioned separately in the experimental part with its manufacturing countries, model, and condition for each tool.
**** We tried rewriting them like you said.
4- In results and discussion there is no equation for the protonation of (SS) like that of AAm and (DMAPAA).

**** We added the equations in Materials.
5- References should be added for the protonation and dissociation of both AAm and (DMAPAA).

**** We added the reference.
6- All equations should be numbered.

**** We did so.
7- NMR not discussed in the manuscript, the authors should add brief discussion.

**** We added the discussion.
8- Figure captions should be added at the end of each provided figure.

**** We have added figure captions except where it is not necessary.
9- In electroplating industries as mentioned in introduction part, where the authors can apply their best hydrogel composition, how they can change the pH of the resulting actual wastewater?

**** We have written about this at the end of the text.

------------------------------------------------------------------------

6. PLOS authors have the option to publish the peer review history of their article ([what does this mean?](https://journals.plos.org/plosone/s/editorial-and-peer-review-process#loc-peer-review-history)). If published, this will include your full peer review and any attached files.
If you choose “no”, your identity will remain anonymous but your review may still be made public.

**Do you want your identity to be public for this peer review?** For information about this choice, including consent withdrawal, please see our [Privacy Policy](https://www.plos.org/privacy-policy).

Reviewer #1: No

Reviewer #2: **Yes:** Prof. A. M. Abdel-Ghaffar

[NOTE: If reviewer comments were submitted as an attachment file, they will be attached to this email and accessible via the submission site. Please log into your account, locate the manuscript record, and check for the action link "View Attachments". If this link does not appear, there are no attachment files.]

While revising your submission, please upload your figure files to the Preflight Analysis and Conversion Engine (PACE) digital diagnostic tool, <https://pacev2.apexcovantage.com/>. PACE helps ensure that figures meet PLOS requirements. To use PACE, you must first register as a user. Registration is free. Then, login and navigate to the UPLOAD tab, where you will find detailed instructions on how to use the tool. If you encounter any issues or have any questions when using PACE, please email PLOS at [figures@plos.org](mailto:figures@plos.org). Please note that Supporting Information files do not need this step.

*In compliance with data protection regulations, you may request that we remove your personal registration details at any time.* [*(Remove my information/details)*](https://www.editorialmanager.com/pone/login.asp?a=r)*. Please contact the publication office if you have any questions.*
